# Supplementary material for: Machine learning approach for automatic recognition of tomato-pollinating bees based on their buzzing-sounds
Source: PLoS Comput Biol. 2021 Sep 16;17(9):e1009426. doi: 10.1371/journal.pcbi.1009426 (PMC8478199; doi:10.1371/journal.pcbi.1009426)
Supplement: S1 Table — (PDF) [file pcbi.1009426.s001.pdf]

S1 Table. Overview of the studies applying Machine Learning and audio feature extraction methods to the acoustic monitoring/detection of bees.

Study	Year	Feature extraction method	ML Classifier	Classification problem	Locality	Dataset type and size	No. of classes
[1]	2016	MFCC	J48 tree, Naïve Bayes, SVM, Random Forest	Classification of bumblebee species and castes	Slovenia	1120 sound samples	12 bumblebees species
[2]	2018	MFCC, STFT, mel-spectrogram, STFT spectral contrast, and tonnetz	LR, KNN, SVM, RF	Distinguishing the bee buzzing sounds from cricket chirping and ambient noise	Logan, USA	Two datasets of 10,260 and 12,914 audio samples	Three categories: buzzing-bees, cricket chirping and ambient noise
[3]	2019	STFT, MFCCs, a method based on Wavelet decomposition and HHT	CNNs and SVM	Detection of an orphaned colony	Marche, Italy	200 minutes of recording sound in colony with its queen and 200 minutes of an orphaned colony	Two classes: beehives with the queen bee present and without the queen bee
[4]	2019	MFCC	SVM	Classification of bee and hornet species	Fukuyama and Kyoto, Japan	480 audio recording samples	Two pollinating bee species (*Apis mellifera* , *Bombus ardens*, *Tetralonia nipponensis*) and a hornet *Vespa simillima xanthoptera*)
[5]	2019	MFCC and HHT	SVM and CNN	Detection of the queen bee presence in honey bees hives	Marche, Italy	576 audio samples/10 minutes duration each	Presence/absence of the queen bee in a hive.
[6]	2019	Goertzel algorithm [7]	SVM	Predict and map the behavior of pollinating flying insects	Antwerp, Belgium	260 audio samples	Three classes: wild bees, bumblebees and background noise.
[8]	2020	MFCC	SVM	Recognition of honeybee hive circadian rhythm	North Poland	3000 audio samples	Two classes: day/night cycles.

Abbreviations: RF= Random Forest, MLP= Multilayer Perceptron, SVM= Support Vector Machines, LR= Logistic Regression, KNN= K-Nearest Neighbors, CNN= Convolutional Neural Networks, GBM= Gradient Boosting Machine, MFCC= Mel frequency cepstral coefficients, STFT= Chroma Short term Fourier Transform, HHT= Hilbert-Huang transform.

References

1. Gradišek A, Slapničar G, Šorn J, Luštrek M, Gams M, Grad J. Predicting species identity of bumblebees through analysis of flight buzzing sounds. Bioacoustics. 2017;26(1):63–76.

2. Kulyukin V, Mukherjee S, Amlathe P. Toward Audio Beehive Monitoring: Deep Learning vs. Standard Machine Learning in Classifying Beehive Audio Samples. Applied Sciences. 2018;8(9).

3. Terenzi A, Cecchi S, Orcioni S, Piazza F. Features Extraction Applied to the Analysis of the Sounds Emitted by Honey Bees in a Beehive. In: 2019 11th International Symposium on Image and Signal Processing and Analysis (ISPA). 45; 2019. p. 03–08.

4. Kawakita S, Ichikawa K. Automated classification of bees and hornet using acoustic analysis of their flight sounds. Apidologie. 2019;50(1):71–79.

5. Nolasco I, Terenzi A, Cecchi S, Orcioni S, Bear HL, Benetos E. Audio-based identification of beehive states. In: ICASSP 2019-2019 IEEE International Conference on Acoustics, Speech and Signal Processing (ICASSP). 4. IEEE; 2019. p. 8256–8260.

6. Van Goethem S, Verwulgen S, Goethijn F, Steckel J. An IoT solution for measuring bee pollination efficacy. In: 2019 IEEE 5th World Forum on Internet of Things (WF-IoT). IEEE; 2019. p. 837–841.

7. Sysel P, Rajmic P. Goertzel algorithm generalized to non-integer multiples of fundamental frequency. EURASIP J Adv Signal Process. 2012;2012:56.

8. Cejrowski T, Szymański J, Logofătu D. Buzz-based recognition of the honeybee colony circadian rhythm. Computers and Electronics in Agriculture. 2020;175:505–486.
