# Supplementary material for: Machine learning approach for automatic recognition of tomato-pollinating bees based on their buzzing-sounds
Source: PLoS Comput Biol. 2021 Sep 16;17(9):e1009426. doi: 10.1371/journal.pcbi.1009426 (PMC8478199; doi:10.1371/journal.pcbi.1009426)
Supplement: S2 Table — Internal numbers correspond to P-values obtained by the T-test; P-values highlighted in bold (p ≤ 0.05) indicate significant differences among the F1-score of the ML algorithms/baselines. (PDF) [file pcbi.1009426.s002.pdf]

S2 Table. Pairwise comparison of the performance of the Machine-Learning algorithms and Baseline scenarios (majority class, Fundamental frequency, and Fundamental frequency (SVM)) in acoustic recognition of bee genera based on buzzing-sounds produced during three behavioral contexts (flight, sonication, and flight + sonication). Internal numbers correspond to P-values obtained by the T-test; P-values highlighted in bold ($p \leq 0.05$) indicate significant differences among the F1-score of the ML algorithms/baselines

**Flight**					
Algorithms	LR	SVM	RF	DTree	Ensemble
LR	–	–	–	–	–
SVM	**0.000**	–	–	–	–
RF	**0.001**	**0.001**	–	–	–
DTree	**0.003**	**0.003**	0.070	–	–
Ensemble	0.144	0.085	0.055	**0.018**	–
Majority class	**0.000**	**0.000**	**0.001**	**0.017**	**0.006**
FF	**0.000**	**0.000**	**0.020**	**0.016**	0.633
FF (SVM)	**0.000**	**0.000**	**0.046**	0.399	**0.049**
**Sonication**					
Algorithms	LR	SVM	RF	DTree	Ensemble
LR	–	–	–	–	–
SVM	**0.004**	–	–	–	–
RF	**0.001**	**0.000**	–	–	–
DTree	**0.000**	**0.000**	**0.008**	–	–
Ensemble	**0.003**	**0.000**	**0.001**	**0.001**	–
Majority class	**0.000**	**0.000**	**0.000**	**0.001**	**0.000**
FF	**0.004**	**0.000**	**0.000**	**0.002**	0.710
FF (SVM)	**0.000**	**0.000**	**0.000**	0.508	**0.000**
**Complete (Flight + Sonication)**					
Algorithms	LR	SVM	RF	DTree	Ensemble
LR	–	–	–	–	–
SVM	0.200	–	–	–	–
RF	**0.030**	**0.000**	–	–	–
DTree	**0.004**	**0.000**	**0.001**	–	–
Ensemble	0.975	**0.045**	**0.001**	**0.000**	–
Majority class	**0.000**	**0.000**	**0.000**	**0.000**	**0.000**
FF	**0.000**	**0.000**	**0.013**	**0.002**	**0.001**
FF (SVM)	**0.001**	**0.000**	**0.000**	**0.031**	**0.000**
