# Supplementary material for: Machine learning approach for automatic recognition of tomato-pollinating bees based on their buzzing-sounds
Source: PLoS Comput Biol. 2021 Sep 16;17(9):e1009426. doi: 10.1371/journal.pcbi.1009426 (PMC8478199; doi:10.1371/journal.pcbi.1009426)
Supplement: S3 Table — Internal numbers correspond to P-values obtained by the T-test; P-values highlighted in bold (p ≤ 0.05) indicate significant differences among the F1-score of the ML algorithms/baselines. (PDF) [file pcbi.1009426.s003.pdf]

S3 Table. Pairwise comparison of the performance of the Machine-Learning algorithms and Baseline scenarios (majority class, Fundamental frequency, and Fundamental frequency (SVM)) in acoustic recognition of bee species based on buzzing-sounds produced during three behavioral contexts (flight, sonication, and flight + sonication). Internal numbers correspond to P-values obtained by the T-test; P-values highlighted in bold ($p \leq 0.05$) indicate significant differences among the F1-score of the ML algorithms/baselines.

**flight**					
Algorithms	LR	SVM	RF	DTree	Ensemble
LR	–	–	–	–	–
SVM	0.218	–	–	–	–
RF	0.094	0.088	–	–	–
DTree	**0.001**	**0.001**	**0.014**	–	–
Ensemble	**0.027**	**0.026**	0.754	**0.002**	–
Majority class	**0.000**	**0.000**	**0.001**	**0.010**	**0.000**
FF	**0.000**	**0.004**	0.052	**0.008**	**0.042**
FF (SVM)	**0.000**	**0.002**	**0.016**	**0.020**	**0.010**
**Sonication**					
Algorithms	LR	SVM	RF	DTree	Ensemble
LR	–	–	–	–	–
SVM	**0.001**	–	–	–	–
RF	0.153	**0.000**	–	–	–
DTree	**0.022**	**0.001**	**0.033**	–	–
Ensemble	0.209	**0.000**	**0.016**	**0.011**	–
Majority class	**0.000**	**0.000**	**0.000**	**0.002**	**0.000**
FF	**0.006**	**0.000**	**0.021**	0.224	**0.001**
FF (SVM)	**0.003**	**0.000**	**0.005**	0.859	**0.000**
**Complete (flight + Sonication)**					
Algorithms	LR	SVM	RF	DTree	Ensemble
LR	–	–	–	–	–
SVM	0.825	–	–	–	–
RF	**0.016**	**0.001**	–	–	–
DTree	**0.003**	**0.000**	**0.000**	–	–
Ensemble	0.722	**0.017**	**0.002**	**0.000**	–
Majority class	**0.008**	**0.000**	**0.003**	**0.007**	**0.000**
FF	**0.008**	**0.000**	**0.003**	**0.007**	**0.000**
FF (SVM)	**0.004**	**0.000**	**0.001**	0.276	**0.000**
