# Supplementary material for: Machine learning approach for automatic recognition of tomato-pollinating bees based on their buzzing-sounds
Source: PLoS Comput Biol. 2021 Sep 16;17(9):e1009426. doi: 10.1371/journal.pcbi.1009426 (PMC8478199; doi:10.1371/journal.pcbi.1009426)
Supplement: Attachment — Submitted filename: PLOSLetter.docx [file pcbi.1009426.s004.docx]

Dear Dr. Dan Stowell,

Please find attached the revised version of the paper entitled *“Machine Learning approach for automatic recognition of tomato-pollinating bees based on their buzzing-sounds”* (PCOMPBIOL-D-21-01039), by A. P. Ribeiro, et al. We would like to thank the reviewers for providing us with detailed comments, which have helped us to improve our work. A revision of the paper has been undertaken and we hope that the enhancements suggested by the reviewers have been satisfactorily fulfilled. We have attached detailed responses to all the reviewers’ comments, as well as the description of the changes made to the manuscript. In order to make the identification of the main performed amendments and updates easier, they have been **highlighted in blue** **font** in the manuscript. We also prepared a clean version of the revised manuscript, as required by the editorial office.

Looking forward to hearing from you.

Sincerely yours,

**José Neiva Mesquita-Neto** (on behalf of the authors).

**Reply to Editors and Reviewers comments**

We are grateful to the reviewers and Editors for their detailed and constructive comments. The manuscript has been carefully revised to fully address the points raised in the reviews. In order to make the identification of the main performed amendments and updates easier, they have been highlighted in blue font in the revised manuscript. In what follows, we discuss how the manuscript has been modified. For enhanced readability, the answers to the reviewers are shown in bold.

**Editors**

A definite issue is data availability: in the submission information, you state that the data are available from https://doi.org/10.1111/1744-7917.12602 but this links to a 2018 journal article which does not seem to offer any raw data. Plos Comp Biol has specific requirements for data availability (see https://journals.plos.org/ploscompbiol/s/submission-guidelines for information). If the data availability issue is not fixed, the article will certainly be rejected.

The same is true of software code: please see https://journals.plos.org/ploscompbiol/s/code-availability

**Authors: We understand the concerns about data availability. Then, we now provided the dataset and software code in a publicly accessible repository (see** [**https://github.com/alisonrib17/bees-tomato**](https://github.com/alisonrib17/bees-tomato)**).**

As Reviewer 2 points out, the application of MFCCs in ML classifiers such as SVM, random forests, or decision trees to classify audio bee samples is not novel. It has been done before, and is now part of the peer-reviewed electronic beehive monitoring literature. (Lines 112-126 are redundant, and MFCCs can be cited to a standard textbook.) A resubmission should more clearly cite and review previous literature that uses MFCCs and machine learning for bee monitoring, even if the monitoring is of hives rather than pollination.

**Authors: We now reviewed and cited the previous literature that uses MFCCs and machine learning for bee monitoring (L 59-64). We also answered Reviewer #2 comments (see the section below).**

The abstract uses some non-standard English, so I also suggest some small improvements to the abstract:

"The bee-mediated pollination" -> "Bee-mediated pollination"

"it becomes primordial" -> "it is important"

"The ML techniques could lead to automate" -> "ML techniques could be used to automate"

**Authors: Ok, we made the suggested changes and reviewed the spelling of English.**

**Reviewer #1**

1. In the paper, Pereira Ribeiro et al. studied the use of machine-learning algorithms to classify bees visiting the tomato flowers. They first collected a dataset containing flight and sonication buzzes of 15 bee species of 8 genera. After isolating the buzzing sounds, they developed a series of ML classification algorithms to work either on a species or on a genus level. They discuss the results in view of advantages and limitations and possibilities for future implementation in an automated tool for crop monitoring.

The paper is clearly written, the methodology is correct, and the results are reasonable, especially in view of the classification metrics of some related studies that the paper cites. The authors are well-aware of the issues related to the unbalanced data samples and I believe they handled it appropriately. In my opinion, the paper represents a substantial contribution to the field of bioacoustics in pollinator science and has promising implications for future studies.

I have some minor comments that the authors may consider.

1. Introduction: Perhaps mention that the pollination of tomatoes in greenhouses is typically done by bumblebees that are reared particularly for the purpose.

**Authors:** **We agree with the reviewer, we mentioned it in the introduction (see L 22-24)**

2. At some points, the reference is shown as (??), for example lines 61, 183, 191, etc.

**Authors:** **This mistake was generated by the separation of the supplementary material from the main text. Now we have solved it.**

3. Acoustic pre-processing. Here, perhaps mention that the flight and sonication buzzes are pronouncedly different in structure, so they can be easily distinguished from the recordings afterwards (as clearly seen in one of the later figures).

**Authors: We agree with the reviewer and mentioned it in the section (see L 123-125).**

4. Line 294: “quite different” … consider using more concise term

**Authors:** **Ok, we replace it for “can be easily distinguished “ (L 305).**

5. Early in the paper you mention that you would compare the ML results with those of conventional statistical methods, but you only cite a previous paper on the same dataset regarding statistical methods. I suggest you mention this the first time you talk about the comparison and write a couple of sentences more of the discussion.

**Authors:** **We agree with the reviewer. In fact, we compared our results with the performance of analyses considering only fundamental frequency as input, instead of conventional statistics. So, we changed "conventional statistics" to "fundamental frequency" throughout the text and limited the comparison to statistical analyses performed by Rosi-Denadai et al. (2018) to the discussion.**

6. As a possible experiment, perhaps it would be interested to construct a binary classifier between the most common species and “others”, if the most common species is so much more abundant. But probably this is beyond the scope of the present paper.

**Authors: This would be an interesting experiment, we even tested it and achieved a higher Accuracy than our current analysis (see** [**https://github.com/alisonrib17/bees-tomato/tree/master/ResultsBinaryClassification**](https://github.com/alisonrib17/bees-tomato/tree/master/ResultsBinaryClassification)**). However, we think that this is beyond the scope of this MS, but we will consider this experiment in further works.**

**Reviewer #2**

Overall, I have enjoyed reading this article but it has serious problems that must be addressed before it is published.

Summary: The objective of the paper is to provide a preliminary demonstration that some ML algorithms can be used in recognizing the taxonomic identity of bees visiting tomato flowers from audio samples of their buzzing sounds.

The authors formulate two hypothesis:

Hypothesis 1: "Due to the high efficiency and accuracy demonstrated by ML tools in automatic sound classification, we expected that these ML algorithms would obtain a greater performance compared to conventional statistics."

Hypothesis 2: "Based on this, we predicted that the buzzing-sounds produced by floral vibrations are more relevant for recognizing bees taxa."

1. Comment 1: It was unclear to me as I was reading the paper what the authors mean by "conventional statistics." In the section "Advantages of Machine-Learning over conventional statistics" they give references to publication [31] and where they state that their analysis "based on fundamental frequency differences, failed to distinguish between most bee species."

Fundamental frequency differences are not conventional statistics. Perhaps, the authors could explain more clearly what they mean by "conventional statistics."

**Authors: We agree with the reviewer. This was also an issue raised by reviewer #1. We actually compared our results with the performance considering only fundamental frequency, instead of conventional statistics (see the changes throughout the main text and supporting information). So, we reviewed and changed "conventional statistics" to "fundamental frequency" throughout the text.**

2. Comment 2: Line 61: Reference is missing in the sentence "Random Forest, Support Vector Machines, and Logistic Regression are the most applied classifiers, and Mel Frequency Cepstral Coefficients is the most used feature extraction strategy (see ??)."

**Authors: This mistake was generated by the separation of the supplementary material from the main text. Now we have solved it.**

3. Comment 3: Lines 91-92: "The acoustic recording of buzzes was carried out using tomato plants (Lycopersicon esculentum ac. BGH 7488) grown at the experimental fields of the Federal University of Viçosa (Minas Gerais, Brazil). A professional hand recorder (SongMeter SM2, Wildlife Acoustics, USA) was used to record the buzzing sounds of bees visiting the tomato flowers."

This is a very insufficient description for me to assess the accuracy of the obtained samples. How were the audio samples obtained? Where was the recorder placed? Was it hand-held? How closely was it placed to the pollinating bees? For a method to be scientific, it must be replicated by other researchers. As a researcher I cannot replicate the data acquisition method of the authors. The authors should provide a picture/drawing of how they recorder was placed with respect to the bee and the tomato plans.

**Authors: We agree with the reviewer, we now provide a more detailed description of how the buzzing sounds were recorded, see L 104-108.**

4. Comment 4: I might have missed it, but I did not see any references to the availability of the authors' dataset, which has a negative impact on replicability.

**Authors: We now provided the dataset in a publicly accessible repository: https://github.com/alisonrib17/bees-tomato**

5. Comment 5: It looks like there were a total of 321 individual bees captured. Are these the bees from which the audio samples were obtained? How many audio files (wav files) were obtained from these bees?

**Authors: The capture of the individual was a requirement to assure the taxonomic identification and to consider the corresponding audio sample on our analysis. Then, the number of individuals captured corresponds to the number of audio collected, especified on Table 1 “N recordings” column. We also added this information to the text (see L 111-115)**

6. Comment 6: Line 104: "Parts with no bee sound or high background noise disturbance were not considered."

This makes the results significantly weaker. This fact should be explicitly mentioned in the abstract. When an audio classifier is deployed in the real world, it must deal with the problem of noise, especially ambient noise such as lawn mowers, cars, human speech, wind, music, etc.

**Authors: We thank the reviewer for this comment. This was a spelling mistake. Actually, we did not exclude the background noise, instead, we kept the audio as it was recorded. We just labeled parts of the audio signal with the type buzzing sounds (sonication or flight) to the algorithm recognition. We also rewrote this sentence (see L 125-126).**

7. Comment 7: Lines 106-107: "The length of individual recordings ranged from five seconds to over one minute."

How were these recordings distributed among the training and testing data?

**Authors: The recordings were distributed according to the amount of samples per species/genera, the temporal variation of the recordings has no relation to the distribution (see L 161-188).**

8. Comment 8: The authors write: "After, the resulting data set was split into 50% for the training/development set (delimited by the red dashed line) and 50% for the testing data set." A more standard split is 70-30? Why 50-50? I guess there is an explanation of sorts in lines 161-166.

**Authors: The distribution of the data was very unbalanced. If we would adopt the 70-30 split, some species might be out of the test set, considering the two classification categories: flight and sonication. Therefore, we needed a split that was able to distribute the data in such a way that there would be audio segments for training and testing, in which case the 50-50 split was the best choice. The subsection "Data split" details this issue (see L 161-188).**

9. Comment 9: Lines 112 -- 126 are redundant. MFCCs are very well know since the 1980's in the audio processing communities. A couple of references are sufficient.

**Authors: The reviewer is right, so we followed his/her suggestion and summarized this part (see L 136).**

10. Comment 10: Lines 134 - 136: "This was necessary because MFCC cannot generate a larger number of features and the average duration of the fragments was small, approximately 1.54 seconds."

This is where I got very confused. In lines 106-107, the authors write: "The length of individual recordings ranged from five seconds to over one minute."

What is the difference b/w an individual recording and a fragment? What do the authors mean by a "fragment"?

**Authors: During the buzzing sound sampling, we followed a bee individual during visits to multiple flowers, as much as possible before stopping audio recording and capturing it. We marked the moment when the segment of buzz started and when it finished, using an audio processing program, as described in Lines 112-117. Consequently, one audio recording corresponds to one individual bee and vice-versa; an audio recording can have segments of the same bee sonicating a flower, flying to another, then sonicating it, and so on. Then, the "fragment" refers to segments of the audio recording (or audio file) with buzzing sounds (sonication or flight). Therefore, an audio file (or sound recording) can be composed of multiple segments of sonication and flight and parts without a buzzing sound (eg. a bee was walking in the flower, cleaning its body, or immobile). To avoid pseudoreplication, we considered the segments belonging to the same audio recording/bee individual as the same replica (recording sample). Moreover, we replaced "fragment" with “segment” to clarify that we did not cut the audio recording, we just classified it into segments of sonication and flight (see L 113-114; Table 1; throughout the main text).**

11. Comment 11: Lines 152-155: "During an exploratory analysis, we detected an unbalance of the sampled data between the classes (species/genera) and between the two behaviors (sonication and flight). There were 103 sound samples referring to flight and 218 samples corresponding to sonication, totaling 321 samples."

So, if my understanding is accurate, 1) there are 321 individual bees (Table 1); 2) there are "fragments" with an average duration of approximately 1.54 seconds (lines 134-136); 3) there are a total 642 audio samples (lines 152-155).

How are these entities related? How are the 642 audio samples obtained? The authors must include another table with a detailed distribution of the samples among the bee tax: number of Augochloropsis brachycephala samples; number of Augochloropsis sp.1 samples; number of Augochloropsis sp.2 samples, etc.

**Authors: The number of bee individuals corresponds to the number of recording samples (N=59), as detailed in our answer to comment 10. Then, the buzz of each recording was manually classified into "sonication" or "flight" by an experienced researcher using audio-processing software. So, the sample size would be the number of audio/sound "recordings", not the "fragments" of buzzing, because one recording corresponds to one bee individual. Moreover, we improved the writing to make this clear in the text (see L 122-123; L 163-164).**

12. Comment 12: Lines 188-191: "Therefore, we combine three classifiers (Random Forest, SVM e Logistic Regression) by majority vote. These classifiers were chosen because they achieved the best performance in recognizing bees buzzing-sounds (see ??)." There is a missing reference in the above sentence.

**Authors: This mistake was generated by the separation of the supplementary material from the main text. Now, we have solved it.**

13. Comment 13: The authors report that the best flight performance metrics of the SVM classifier in Table 2 are 55.76, 56.91, 53.78, 49.00. These are weak by ML standards on such a small dataset. The sonification metrics of the SVM are better: 73.39 61.75 60.70 59.06, but are still very low, which suggests to me one of the three things:

1) the SVM, although the best among the reported classifiers, is not a good fit for this domain;

2) the MFCC features are either inappropriate or insufficient for this domain;

3) there is so little audio data that no classifier can, in principle, generalize well on it.

I came to the same conclusions when going through the data in Table 3. The authors should address these points both in the abstract and the discussion.

**Authors: We thank the reviewer for this comment. We addressed these issues in the discussion (see L 429-431); we now highlight the issues regarding the little data set as a possible explanation to the low performance of our analysis in relation to ML standards (see L 432-435; Abstract).**

14. Comment 14: Lines 317-318: "Just the LR and SVM classifiers always presented Macro-F1 values higher than the baselines at genus-level classification (??)." There is a missing reference in the above sentence.

**Authors: This mistake was generated by the separation of the supplementary material from the main text. Now we have solved it.**

15. Comment 15: Lines 319-321: there are two missing references.

**Authors: This mistake was generated by the separation of the supplementary material from the main text. Now we have solved it.**

16. Comment 16: Lines 336-337: "The ML algorithms were capable of recognizing most of the flower-visiting bees of the tomato crop, based only on the characteristics of their buzzing sounds."

This is a very vague statement, and a misleading one. A more accurate description would be that the tested ML algorithms' accuracy ranged from 49 to 74% on a dataset of 642 audio samples with the audio samples with ambient noise removed from the dataset.

**Authors: We agree with the reviewer's comment and rephrased this sentence (see L 348-349).**

17. Comment 17: Lines 358-362: "Moreover, the ML combined with the MFCC method has been able to correctly predict 66% of all samples; 79% of the samples of species based on sonication sounds and SVM algorithm. Likely due to the ML attributes and boosted by MFCC, we reached here higher performance on acoustic recognition of bees than the conventional statistics, thus, corroborating our hypothesis 1."

18. Comment 18: The authors never made clear what they mean by "conventional statistics." What exactly is being compared? The comparisons from [31] should be briefly summarized in this article? What were they? Are they significantly better than the results reported in this article? Otherwise, there is little evidence corroborating hypothesis 1.

**Authors: Comments 17 and 18 seem to be part of the same query, so we answer both of them here. We agree with the reviewer, we actually compared the ML classification powered by the MFFC with the classification based on the fundamental frequency alone. Rosi-Denadai et al. (2018), based on statistical analyses, found no differences among the fundamental frequency of the visiting bees. So, we rephrase this section and check the overall text to clarify it. Further, we excluded the sentence about corroborating hypothesis 1.**

19. Comment 19: Many references are missing in the section "Advantages and limitations of buzzing-sound classifications with Machine Learning."

**Authors: This mistake was generated by the separation of the supplementary material from the main text. Now we have solved it.**

20. Comment 20: Lines 381-382: "Therefore, corroborating our hypothesis 2 only for species recognition."

There is insufficient evidence to corroborate this hypothesis. This sentence should be either reworded or removed from the article altogether.

**Authors: Ok, we now have removed this sentence from the article.**

**Reviewer #3:**

1. The manuscript ID PCOMPBIOL-D-21-01039 entitled "Machine Learning approach for automatic recognition of tomato-pollinating bees based on their buzzing-sounds" is an interesting and novel contribution about taxa recognition of bees which buzz-pollinated the tomato using buzzing sounds. However, it is not very clear why it is importance that farmers or agronomists know the taxonomical identification of buzzing bees. All buzzing bee could be consider efficient pollinators of tomato? In other solanaceous plants if bees is too small only function as thief or if is too big could damage the flower. Authors need to review more literature to justify better the use of this application. Does this ML is possible to use with other buzz-pollinated plants? It will be interesting to discuss this possibility. This application could be very useful to buzzing bee identification in other buzz-pollinated plants.

**Authors: We thank the reviewer for the valuable comments. . We now dedicated a section to discuss the relevance of our results to farmers and the applicability to other buzz-pollinated plants (see L 437-461).**

2. Also, I included the following minor’s corrections:

Please review the actual scientific name of tomato. It seems that the actual is Solanum lycopersicum L. and the synonym is Lycopersicon esculentum Mill.

**Authors: We followed the classification of Tropicos.org, which is specialized in plants nomenclature, where the accepted name is *Lycopersicon esculentum* (see https://www.tropicos.org/name/29602513), but according to GBIF Secretariat the scientific name of tomato is *Solanum lycopersicum* L. (https://www.gbif.org/es/species/2930137). Therefore, there does not seem to be a consensus on the scientific name. However, we replaced *Lycopersicon esculentum* with *Solanum lycopersicum* because it's a well-known name.**

3. Please review “Augochloropsis” spelling in Table 4 and along the paper.

**Authors: We thank the reviewer for noting this, we checked the spelling of “Augochloropsis” throughout the text.**

4. L61, 183, 191, 318, 320, 321, 395, 418. Please check “??”

**Authors: This mistake was generated by the separation of the supplementary material from the main text. Now we have solved it.**
